# Natural and Eco-Friendly Materials for Triboelectric Energy Harvesting

**DOI:** 10.1007/s40820-020-0373-y

**Published:** 2020-01-28

**Authors:** Vladislav Slabov, Svitlana Kopyl, Marco P. Soares dos Santos, Andrei L. Kholkin

**Affiliations:** 1 Department of Physics and CICECO-Aveiro Institute of Materials, University of Aveiro, 3810-193 Aveiro, Portugal; 2 Centre for Mechanical Technology and Automation (TEMA), University of Aveiro, 3810-193 Aveiro, Portugal; 3 Department of Mechanical Engineering, University of Aveiro, 3810-193 Aveiro, Portugal; 4 School of Natural Sciences and Mathematics, Ural Federal University, Ekaterinburg, Russia 620000; 5 Laboratory of Functional Low-Dimensional Structures, National University of Science and Technology MISiS, Moscow, Russia 119049

**Keywords:** Natural and eco-friendly materials, Energy harvesting, Triboelectric nanogenerators, Biocompatibility

## Abstract

Triboelectric nanogenerators (TENGs) are promising electric energy harvesting devices as they can produce renewable clean energy using mechanical excitations from the environment. Several designs of triboelectric energy harvesters relying on biocompatible and eco-friendly natural materials have been introduced in recent years. Their ability to provide customizable self-powering for a wide range of applications, including biomedical devices, pressure and chemical sensors, and battery charging appliances, has been demonstrated. This review summarizes major advances already achieved in the field of triboelectric energy harvesting using biocompatible and eco-friendly natural materials. A rigorous, comparative, and critical analysis of preparation and testing methods is also presented. Electric power up to 14 mW was already achieved for the dry leaf/polyvinylidene fluoride-based TENG devices. These findings highlight the potential of eco-friendly self-powering systems and demonstrate the unique properties of the plants to generate electric energy for multiple applications.

## Highlights

- An up-to-date review of the natural materials used for triboelectric energy harvesting is provided.
- Major parameters of the electric output are identified and compared for different materials.
- Best results (14 mW) were obtained for dry leaf powder in combination with poly(vinylidene fluoride) in contact-separation mode.

## Graphical abstract

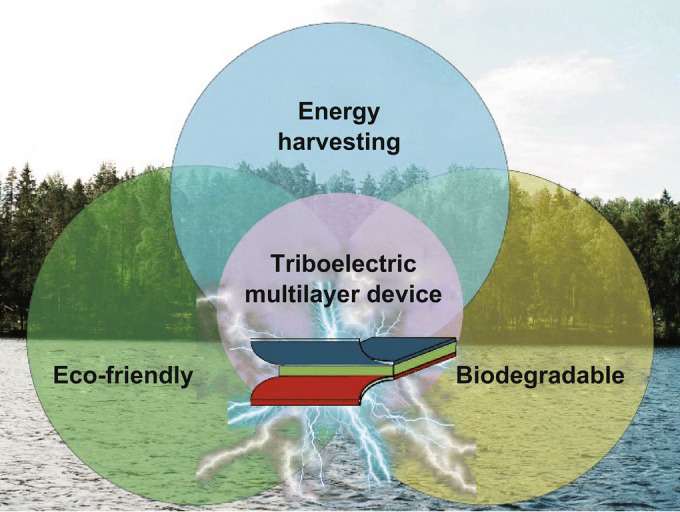

## Introduction

Energy harvesting is a global area of research that includes the development of suitable methods of environmental energy collection, selection of materials, and customization of harvester designs, so that harvesters can operate over long periods of time with reduced intermittency and impact on environment, while ensuring low production and ecological costs. Many transduction mechanisms for energy harvesting have already been developed, and many designs of harvesting devices have been provided for a wide range of applications from small- to large-scale powering [1–7]. A few years ago, the interest of researchers was attracted by the triboelectric effect, by which electricity is produced due to mechanical friction between two bodies in a motion [8, 9]. This triboelectric effect has been known for a long time and is thought to be purely electrostatic in binary systems [10, 11]. Charge generation from friction has also been investigated in some polymers due to the chemical reaction of radicals [12, 13]. Though triboelectricity has been observed a long time ago, it was considered as a harmful phenomenon and many efforts were spent to avoid it (e.g., to facilitate the transport of powders and recycled materials) [14–17]. Only recently triboelectricity was applied for self-powering of a broad range of highly sophisticated devices, although the charge generation mechanism is still under discussion [18, 19]. However, despite the absence of the viable theory of triboelectricity, such findings opened up new possibilities for electric energy harvesting [20–22].

**Fig. 1:**
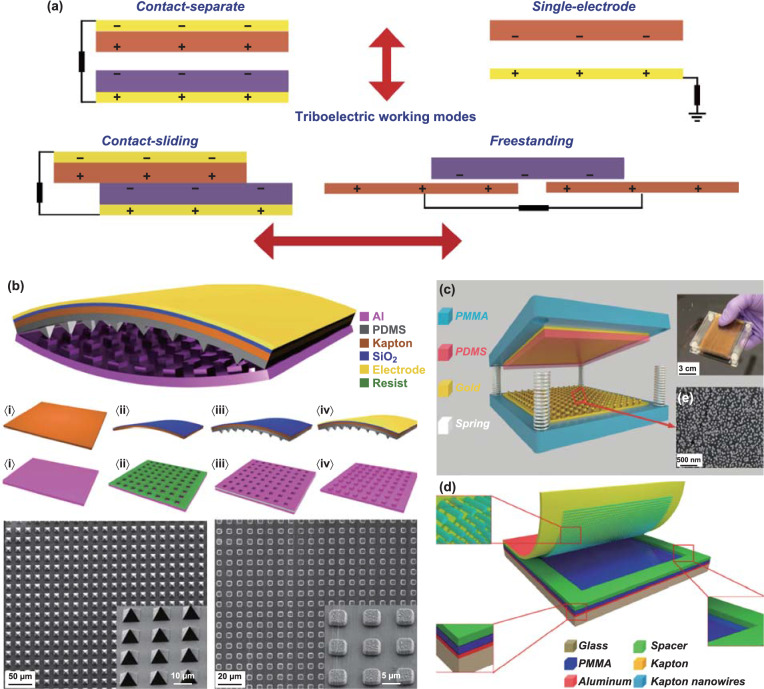
The main modes of triboelectric nanogenerators (TENGs) and examples of construction: **a** four main working modes, and **b** example of the surface modification of the polymer-based TENG. Reprinted with permission from Ref. [30]. Copyright (2012) American Chemical Society. **c** Example of construction of a TENG operating under the contact-separation mode using springs. Adapted with permission from Ref. [36]. Copyright (2013) American Chemical Society. **d** Schematic illustration of the polymer TENG operating under the contact-separation mode using a spacer. Reprinted with permission from Ref. [39]. Copyright (2012) American Chemical Society

As the charge generation phenomena occur mainly on the nanoscale, energy harvesting devices based on triboelectric effect are called triboelectric nanogenerators (TENGs). Four work modes have already been identified to characterize and apply TENGs for electric energy harvesting: vertical contact-separation mode, lateral sliding mode, single-electrode mode, and freestanding triboelectric layer mode (Fig. 1a) [23]. During the motion process (sliding or separation), opposite charges on the surface of materials are produced, whose amount will significantly increase if the surface area increases and changes according to the different composition and structure of materials [24, 25]. Several studies have been conducted to understand the charge formation mechanisms, and they also provided the basis for measurements standards [26–28]. In another work, Zou et al. [29] demonstrated how to measure triboelectricity by using several polymers as examples. Since the most used materials for TENGs are currently polymers, their surfaces were modified to increase the output electric current and voltage (Fig. 1b) [30–33]. Moreover, the effect of surface functionalization on the main features of TENG harvesters has been rigorously studied. As a result, many materials with the ability to harvest energy by friction have been identified [34, 35]. In addition, several efficient designs for applications using powering by TENG harvesters have been proposed (Fig. 1b–d) [36–39]. A common architecture of TENGs designed according to the contact-separation mode uses springs or polymer spacers between the bodies [4, 37].

Recently, several natural materials were found with ability to harvest electric energy from dynamics of mechanical power sources ensuring low-cost production, as well as ecological safety and biocompatibility during uninterrupted or intermittent contacts with the human body. Humans may provide a large amount of mechanical energy during their daily living (the electric power may exceed 70 W), which can be converted to produce significant amounts of useful energy by triboelectric generation [40, 41]. Many applications (e.g., innovative biomedical sensors that monitor daily activity and functioning of the organism and multifunctional medical devices) require biocompatible materials in contact with human skin or implanted inside the body [42–44]. Typically, the materials for biocompatible applications are those produced by nature or using natural products [45–47]. This paper presents an up-to-date review of the relevant literature reports that highlight major scientific achievements in this area and guides readers through innovative approaches for power generation, harvester designs and parameters that influence the output performance. Our main goal is to provide the relevant findings related to simple materials produced by nature that can substitute fully polymeric TENGs such that they can be used to produce a significant amount of electric energy via the triboelectric effect.

## Natural Materials

Natural materials are a special class of materials that can be suitably used for triboelectric applications. By definition, this class is fully biocompatible and eco-friendly without any recycling problems. These materials opened up a new possibility for harvesting electric energy because their manufacture is neither complex nor expensive, and their output performance is similar to traditional polymer/ceramic materials. TENGs based on natural materials have been recently investigated in detail, and the conclusions showed that their complex surface architecture causes major impacts on the produced charge, current, or voltage [48–51]. Since natural materials have hierarchically structured surfaces, surface modification is not required in many cases, and they can be used for polymer modification by soft lithography as well [52–54]. Besides, the combination of natural materials with biocompatible polymers allows the production of eco-friendly triboelectric pairs for energy harvesting. We highlight here three kinds of biomaterials applied in energy harvesting: (1) plants and their parts (mainly leaves); (2) the plant products, namely sugar and cellulose [55, 56]; (3) biodegradable materials (spider silk, egg white, chitin, etc.). Their harvesting performance and possible applications will be overviewed as well.

### Plants

**Fig. 2:**
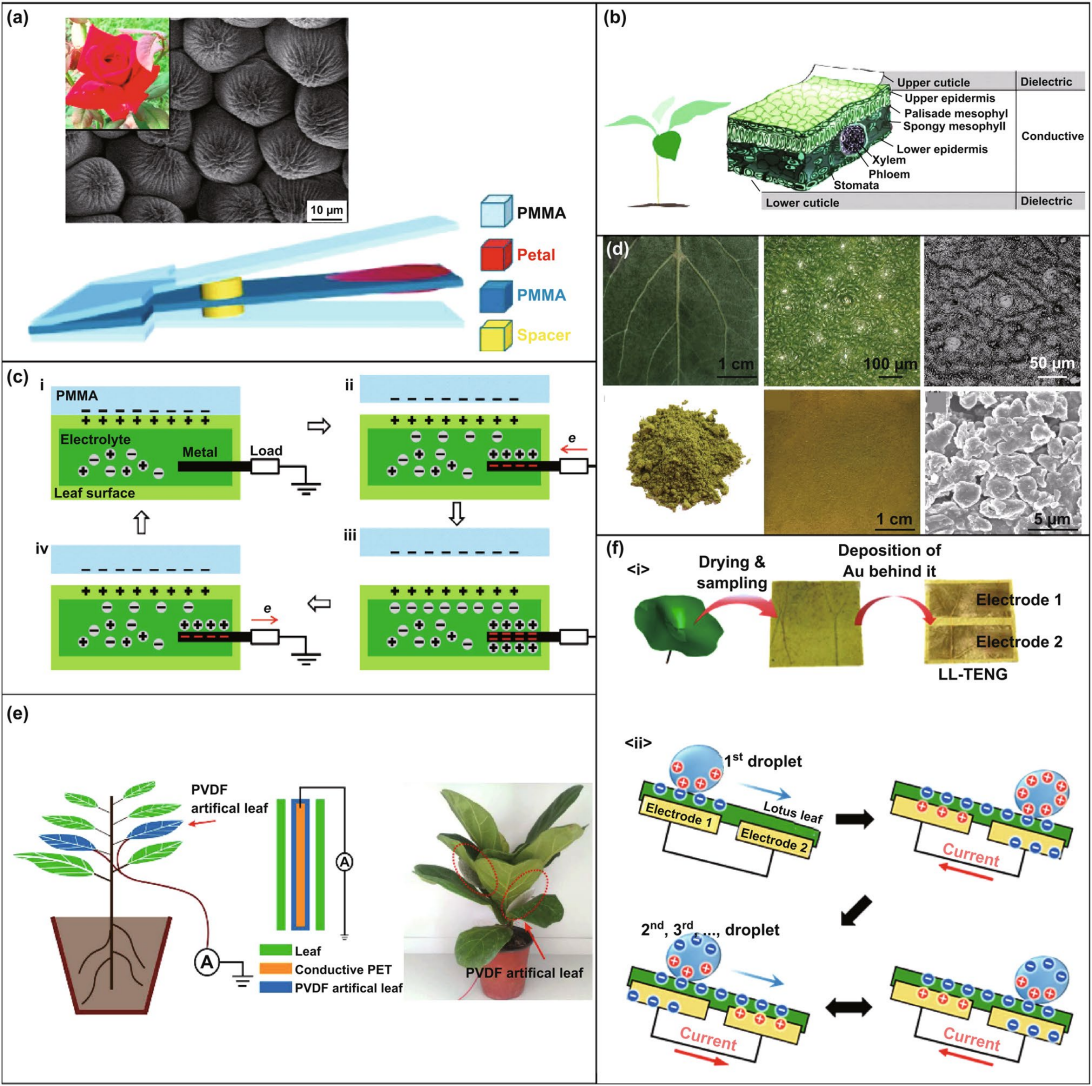
The design of TENGs using plants: **a** surface of the petal rose and schematics of the contact-separation mode TENG with petal. Reprinted with permission from Ref. [60]. Copyright (2018) Published by Elsevier Ltd. **b** Schematics of the rhododendron leaf as an electric bilayer structure. Reprinted with permission from Ref. [61]. Copyright (2018) Wiley-VCH Verlag GmbH & Co. KGaA, Weinheim. **c** Schematic illustration of the working principles of leaf TENG in the single-electrode mode. i) contact state, ii) separating state, iii) separated state, iv) approaching state. Reprinted with permission from Ref. [62]. Copyright (2018) Wiley-VCH Verlag GmbH & Co. KGaA, Weinheim. **d** Structure of the fresh leaf and dry powder. **e** Schematics and proof of the concept of plant modification for designing TENGs. Reprinted with permission from Ref. [63]. Copyright (2018) Published by Elsevier Ltd; **f** Schematics of the water–solid contact. Reprinted with permission from Ref. [64]. Copyright (2017) Published by Elsevier Ltd

As it is well known, the triboelectric effect is based on converting kinetic energy into electric energy during the contact of two different materials in an area-dependent manner [49]. For this reason, materials in contact must have large enough contact areas so that the harvested energy can be maximized [48, 57, 58]. Plant materials provide a wide range of natural surfaces, which allow their application as friction bodies. The surfaces of the plants are, therefore, used as masks for soft lithography—for example, polymers can replicate all surface jaggies of the leaves [59]. Moreover, they can be also used as triboelectric pairs. It is worth to note that triboelectrification occurs naturally in many plants (e.g., in petals) [60], which were already applied to increase friction. The petal surface is characterized by a sophisticated microstructure, which naturally enlarges the contact area between the surfaces (Fig. 2a). Using these properties, a TENG using petals (Chinese rose) was able to supply commercial LEDs by vibration stress [60]. Not only flower petals can be used as natural harvesters based on the triboelectric effect, but also other parts of the plants as well. Noticeably, an extended electric bilayer sub-structure was found in the *Rhododendron* plant and its leaf was also used for triboelectric energy harvesting [61]. Figure 2b presents the schematics of the layers in a *Rhododendron* leaf. Recent findings clearly show that separate parts of leaf body can work as electrical structures, although they must be in contact with other dielectric materials (e.g., polymers) for electric power maximization. (Contact of similar nature materials commonly provides lower output performances.) The use of a plant leaf as a triboelectric pair material was also studied for the case of a *Hosta* leaf [62]. As the natural leaf of any plant is full of water and comprises an electrolyte, it can be also used as a conductive liquid. Figure 2c demonstrates how the electrolyte allows the use of a leaf in a single-electrode mode for triboelectric energy harvesting. Table 1 summarizes the properties of seven different leaves with different natural surfaces. These TENGs clearly demonstrate the impact of the natural materials’ morphology on the output performance. Because the fresh leaf will dry during triboelectric cycling, both options (fresh and dry leaves) have been explored by Feng et al. [63]. These authors [63] designed a hybrid wind–TENG (WTENG) based on the fresh leaf and powder from the dry leaf (Fig. 2d). In this experiment, the dry leaf was milled into a powder and then covered by polylysine (PLL) using a spin-coating method. To compare this modified WTENG with the one based on a leaf, contact-separation mode driven by an electric motor was used. Enhanced electric energy was harvested for the leaf powder: Up to 60 μA and 1000 V under 5 Hz speed frequency were observed. Besides, up to 70 μA and 150 V were achieved using a potted-plant-based wind-driven harvesting system (Fig. 2e) at 7 m s^−1^ wind speed. Combination of WTENG with the tree was intended for several novel applications [62]. Using this design, the leaf TENG can work in a freestanding mode, which allows a transduction of mechanical energy without grounding. The mechanical force can be delivered to the plants either by wind or rain. This fact motivated further research using a similar TENG design based on a *lotus* leaf for water–solid contact scenarios [64] (Fig. 2f).

**Table 1:** Triboelectric pair materials and their triboelectric characteristics

Triboelectric pair: material 1	Triboelectric pair: material 2	Used mode and fabrication methods	Contract area (cm^2^)	Electric characterization^a^	Mechanical excitation
Petal rose [60]	Poly(methyl methacrylate) (PMMA)	Single-electrode mode Handmade	3 × 3	*V*_oc_ = 30.6 V *I*_sc_ = 0.78 µA *P* = 24 µW *P*_density_ ≈ 2.7 µW cm^−2^	100 N 2 Hz
Hosta leaf [62]	Poly(methyl methacrylate) (PMMA)	Single-electrode mode Handmade	8 × 8	*V*_oc_ = 230 V *I*_sc_ = 9.5 µA *P* = 2185 µW *P*_density_ ≈ 34.1 µW cm^−2^	2 Hz 0.333 m/s
M. denudate [62]	Poly(methyl methacrylate) (PMMA)	Single-electrode mode Handmade	8 × 8	*V*_oc_ ≈ 100 V *I*_sc_ ≈ 3.5 µA *P* = 350 µW *P*_density_ ≈ 5.5 µW cm^−2^	2 Hz 0.333 m/s
L. chinense [62]	Poly(methyl methacrylate) (PMMA)	Single-electrode mode Handmade	8 × 8	*V*_oc_ ≈ 110 V *I*_sc_ ≈ 4 µA *P* = 440 µW *P*_density_ ≈ 6.9 µW cm^−2^	2 Hz 0.333 m/s
Firmiana [62]	Poly(methyl methacrylate) (PMMA)	Single-electrode mode Handmade	8 × 8	*V*_oc_ ≈ 90 V *I*_sc_ ≈ 2.8 µA *P* = 252 µW *P*_density_ ≈ 3.9 µW cm^−2^	2 Hz 0.333 m/s
Populus [62]	Poly(methyl methacrylate) (PMMA)	Single-electrode mode Handmade	8 × 8	*V*_oc_ ≈ 115 V *I*_sc_ ≈ 3.5 µA *P* = 402.5 µW *P*_density_ ≈ 6.3 µW cm^−2^	2 Hz 0.333 m/s
Lotus [62]	Poly(methyl methacrylate) (PMMA)	Single-electrode mode Handmade	8 × 8	*V*_oc_ ≈ 100 V *I*_sc_ ≈ 2.8 µA *P* = 280 µW *P*_density_ ≈ 4.4 µW cm^−2^	2 Hz 0.333 m/s
E. aureum [62]	Poly(methyl methacrylate) (PMMA)	Single-electrode mode Handmade	8 × 8	*V*_oc_ ≈ 90 V *I*_sc_ ≈ 2 µA *P* = 180 µW *P*_density_ ≈ 2.8 µW cm^−2^	2 Hz 0.333 m/s
Rhododendron leaves [61]	Ecoflex (silicone elastomer film)	Single-electrode mode Handmade	4.5 × 4.5	V ≈ 140 V *P*_density_ = 15 µW cm^−2^	10 Hz 0.9 N
Fresh leaf [63]	Poly(vinylidene fluoride) (PVDF)	Contact-separation mode Spin-coating + handmade	4 × 4	*V*_oc_ ≈ 430 V *I*_sc_ ≈ 15 µA *P* = 6450 µW *P*_density_ ≈ 403.1 µW cm^−2^	5 Hz
Dry leaf (powder) [63]	Poly(vinylidene fluoride) (PVDF)	Contact-separation mode Spin-coating + handmade	4 × 4	*V*_oc_ ≈ 560 V *I*_sc_ ≈ 25 µA *P* = 14,000 µW *P*_density_ ≈ 875 µW/cm^2^	5 Hz
Polydimethyl siloxane + cellulose nanocrystal flakes (PDMS/CNCFs) [70]	Aluminum	Single-electrode mode Spin-coating + handmade	1.5 × 1.5	*V*_oc_ ≈ 320 V *I*_sc_ ≈ 5 µA cm^−2^ *P* = 1600 µW cm^2^	40 N
Cellulose paper [71]	Poly(caprolactone)/graphene oxide 4% (PCL/GO 4%)	Contact-separation mode Electrospinning + handmade	2 × 4	*V*_oc_ ≈ 120 V *I*_sc_ ≈ 4 µA *P* = 480 µW *P*_density_ ≈ 60 µW cm^−2^	15 N 3 Hz
Cellulose nanofibril paper + 4% Phosphorene [72]	Polyethylene terephthalate (PET)	Contact-separation mode Electrode sputtering + handmade	–	*V*_oc_ = 5.2 V After 6 months *V*_oc_ = 4 V	70 N
Cellulose nanofibrils (CNF) [82]	Fluorinated ethylene propylene (FEP)	Contact-separation mode Handmade	1 × 1	*V*_oc_ ≈ 5 V *I*_sc_ ≈ 7 µA *P* = 35 µW *P*_density_ ≈ 35 µW cm^−2^	10 Hz
			40 × 40	*V*_oc_ ≈ 32.8 V *I*_sc_ ≈ 35 µA *P* = 1148 µW *P*_density_ ≈ 0.72 µW cm^−2^	
Polydimethylsiloxane sponge with sugar assist [86]		Freestanding mode Soft lithography + hand made	–	*V*_oc_ ≈ 450 V *I*_sc_ ≈ 0.04 µA cm^−2^ *P*_density_ = 18 µW cm^−2^	400 N 5 Hz
Air-permeable paper-based TENG [91]		Contact-separation Dip-coating + electrospinning + handmade	4 × 4	*V*_oc_ ≈ 197 V *I*_sc_ ≈ 16.2 µA cm^−2^ *P* = 3191 µW cm^−2^ *P*_density_ = 797.85 µW cm^−2^	85 kPa
Egg white (EG) [89]	Rice paper (RP)	Contact-separation Handmade	1 × 2	*V*_oc_ ≈ 55 V *I*_sc_ ≈ 0.6 µA cm^−2^ *P* = 33 µW cm^−2^ *P*_density_ = 16.5 µW cm^−2^	–
EG [89]	Chitin	Contact-separation Handmade	1 × 2	*V*_oc_ ≈ 8 V *I*_sc_ ≈ 0.8 µA cm^−2^ *P* = 6.4 µW cm^−2^ *P*_density_ = 3.2 µW cm^−2^	–
Rice paper [90]	Polyvinyl chloride PVC	Contact-separation Brush coating + handmade	3 × 3	*V*_oc_ ≈ 392 V *I*_sc_ ≈ 16.7 µA cm^−2^ *P*_density_ = 82.69 µW cm^−2^ Resistance = 80 Ω	5 Hz
Recombinant spider silk [97]	PET	Contact-separation Spin-coating + inkjet water lithography + handmade	48	*V*_oc_ ≈ 2600 V *I*_sc_ ≈ 480 µA cm^−2^ *P*_density_ = 5290 µW cm^−2^	20 N

^a^The peak value was used for the electric characterization

The TENGs using leaves are typically produced by hand, which is a problem for mass production and also limits their future use. However, the possibility of harvesting electric energy by plants highlights the idea of using natural triboelectric processes and renders a deeper understanding of the triboelectric effect designed by nature. The following section is focused on other possibilities to use natural materials, namely via by-products of plant processing. In these cases, the manufacture of TENGs can be carried out by additive technologies and liquid deposition, due to their adaptation capability of these methods.

### Products of Plants Processing

**Fig. 3:**
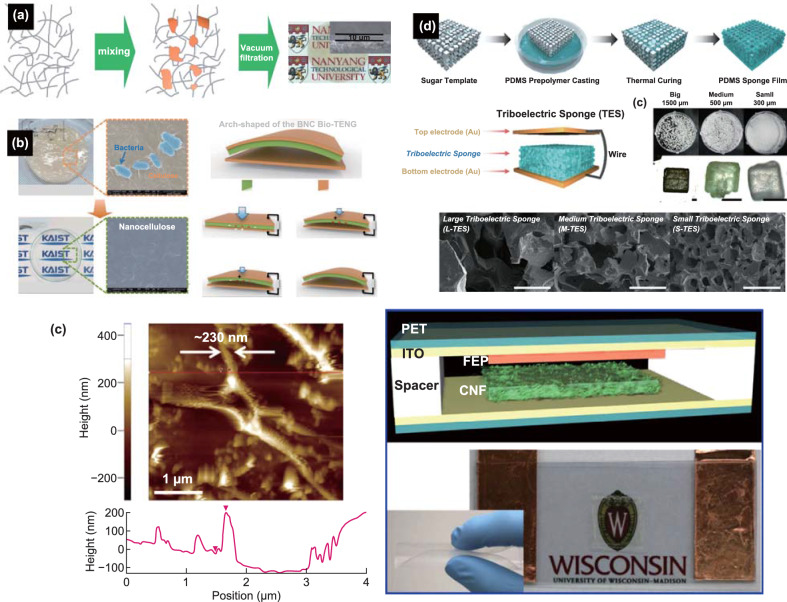
Products of processing of plants used to design TENGs: **a** cellulose/phosphorene hybrid TENG preparation and transparency demonstration. Reprinted with permission from Ref. [72]. Copyright (2017) Wiley-VCH Verlag GmbH & Co. KGaA, Weinheim. **b** Demonstration of a bacterial nanocellulose-based TENG. Reprinted with permission from Ref. [78]. Copyright (2017) Published by Elsevier Ltd. **c** Surface structure, schematic illustration and demonstration of the transparency of a TENG based on cellulose nanofibrils. Reprinted with permission from Ref. [82]. Copyright (2016) Published by Elsevier Ltd. **d** Sponge TENG fabricated by 3D soft lithography using sugar particles. Reprinted with permission from Ref. [86]. Copyright (2016) Wiley-VCH Verlag GmbH & Co. KGaA, Weinheim

Natural materials, such as cellulose and sugar, can be fabricated from plants and used for harvesting electric energy by the triboelectric effect. Both materials have intrinsic piezoelectric properties, which can be applied in hybrid harvesting systems [51, 65] that exploit both triboelectric and piezoelectric effects, such as biomedical sensors and actuators. Currently, the industrial production of these materials is well established. For example, cellulose fibers that are being used for paper manufacturing hold potential for energy harvesting applications [66, 67], as they are readily available and can be used for designing flexible devices [68]. Recently, Alam and Mandal [69] implemented a piezoelectric generator by mixing cellulose nanofibers and polydimethylsiloxane (PDMS) (as a matrix for cellulose) and observed the ability to produce up to 30 V and 500 nA by human-hand punching. As such, this material can be used in triboelectric generators or work as a triboelectric pair due to its high surface area [62]. Following this work, Peng et al. [70] constructed a TENG based on PDMS matrix, in which cellulose nanocrystals were used as fillers. This TENG was fabricated by the spin-coating method using an aluminum (Al) film as electrode. By performing tests in contact-separation mode and comparing its performance with a pure PDMS film with the same thickness, cellulose TENG provided voltages up to ~ 350 V, which correspond to a 10-fold increase as compared to pure PMDS. As pure cellulose can also be used for eco-friendly triboelectric generation, Parandeh et al. [71] proposed to use a cellulose paper as a triboelectric pair material with coated graphene oxide. They fabricated a book-shaped TENG working in the contact-separation mode, which was able to generate up to 120 V [71]. Hybrid cellulose paper was also used by Cui et al. [72]. In this study, cellulose nanofibril–phosphorene transparent paper of 10 µm thickness was fabricated (Fig. 3a). The TENG was designed using a polyethylene terephthalate (PET) film as a triboelectric pair. Mixing of cellulose nanofibrils with phosphorene emphasized the superior performance of this TENG (in comparison with pure cellulose nanofibrils) and protection of the phosphorene against oxidation. In addition, the TENG with a cellulose paper structure showed a decrease in electric output during fatigue tests (20% lower performance after 6 months). Since the stability of the natural materials constituting TENGs is an important feature for most applications, these tests must be always performed to characterize materials. In the past, cellulose was already used in TENGs as a part of an organic–inorganic composite. Shi et al. [73] fabricated a cellulose aerogel with BaTiO_3_ nanoparticles as fillers and coated it using a PDMS layer. The implemented flexible hybrid PENG-TENG nanogenerator was able to produce up to 48 V. Aerogels were also used in the harvester architecture (e.g., TENGs based on bacterial cellulose) [74]. Bacterial cellulose is produced by bacteria, specifically from the *Acetobacter xylinum*, as known since 1886 [75]. This material has been explored for self-powering systems based on piezoelectric transduction [76, 77]. Bacterial nanocellulose was used to fabricate a flexible, transparent and (even more important) bio- and an eco-friendly triboelectric nanogenerator [78] (Fig. 3b). Using a regenerated bacterial nanocellulose (BNC) film, such TENG was prepared via solubilization process from bacterial cellulose, which was in turn produced in a gel state by *Acetobacter xylinum* KJ1 in the Glu-Fruc medium. The performance of this TENG was sufficiently high: accumulative charge of ~ 8.1 µC m^−2^ and peak-power density of ~ 4.8 µW m^−2^. Moreover, Kim et al. [78] carried out additional characterization of the charge transfer by measuring the surface energy of materials under friction. The surface with lower surface energy is negatively charged after friction contact [79–81]. Such characterization complements regular investigations of TENGs and allows deeper insight into the process of charge generation. Yao et al. [82] developed a TENG from thin transparent film based on cellulose nanofibers (CNF). In this work, a triboelectric pair was implemented using fluorinated ethylene propylene (FEP) (Fig. 3c). Moreover, the influence of the surface area on the TENG output performance was found. A significant increase in the open-circuit voltage was observed when the area was increased from 1 to 40 cm^2^ (spacer of 0.1 cm thickness). Such a TENG with the area of 1 × 1 cm^2^ was able to provide almost the same performance as standard polymer TENGs based on Kapton–PET and polytetrafluoroethylene (PTFE)–polyamide materials [18, 83].

Sugar is also a simple natural material that exhibits both piezoelectric and triboelectric properties, which are quite useful for energy harvesting applications [84]. Chun et al. [85] developed a sponge TENG based on PDMS with embedded Au nanoparticles. This sponge TENG was designed to work in a contact-separation mode. This work presented the novel approach to simplify the fabrication process based on replacement of a spacer by the pores. As sugar can be easily dissolved in water, it can be further explored using the soft lithography method. Kim et al. [86] manufactured the sponge TENG based on the sugar-polydimethylsiloxane composite, so that it was able to perform in a freestanding mode (Fig. 3d). The thin sponge-based TENG was tested using three different particles sizes. It turned out that the output characteristics were able to be controlled by the sugar filling and pore size. By changing the structure from flat to porous, it was possible to increase the output performance by five times, thus highlighting the effect of the sugar. Triboelectric transduction was also strongly influenced by the contact area and corresponding pore size. Filling the sponge with smaller particle size of the sugar seems to result in higher output characteristics. Park et al. [87] also highlighted the ability of this technology to produce a highly efficient TENG. A similar sponge TENG was engineered using sugar filling with the addition of ferroelectric nanoparticles [87].

### Natural Biodegradable Materials

The first biodegradable triboelectric nanogenerator (BD-TENG) for short-term biomechanical energy conversion in vivo was developed in 2016 [88]. Due to the design of a multilayer structure, consisting of biodegradable polymers (BDP) and absorbable metals, this BD-TENG is able to decompose and dissolve in the animals’ body after the working cycle without any adverse long-term effect. Based on these results, fully bioabsorbable triboelectric nanogenerators (BN-TENGs) performing in vivo were developed by Jiang et al. [89]. A comprehensive research of “triboelectric series” of five natural bioresorbable materials was conducted by testing pairwise combinations, which significantly promoted the development of natural materials for TENGs and other triboelectric devices.

**Fig. 4:**
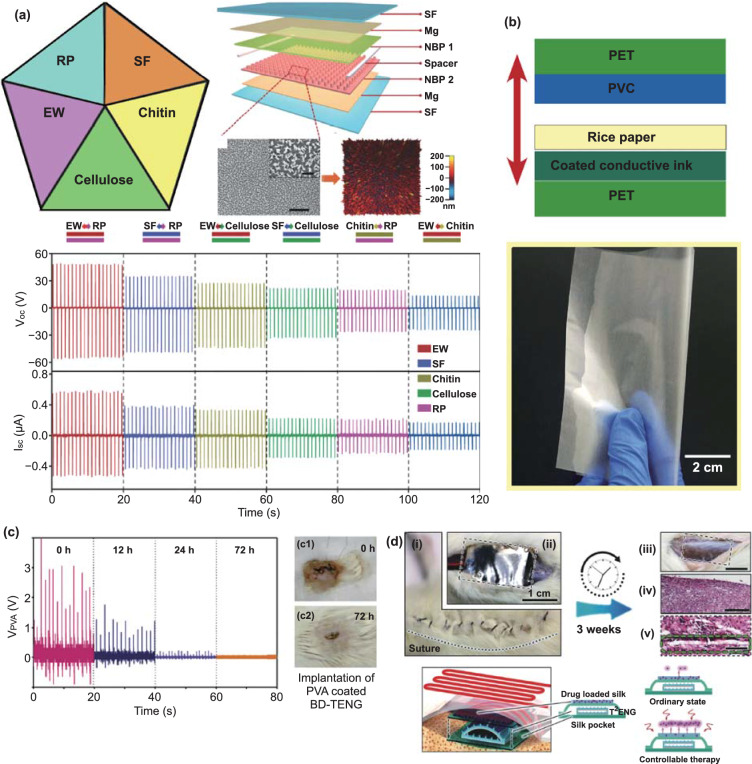
**a** NBPs obtained from wide raw material sources and structure diagram of a typical BN-TENG device: scanning electron microscopy (SEM) and atomic force microscopy (AFM) images of nanostructure on the surface of NBP film. Lower and upper scale bars: 5 and 1 µm. Reprinted with permission from Ref. [89]. Copyright (2018) Wiley-VCH Verlag GmbH & Co. KGaA, Weinheim. **b** Photograph of the rice paper and scheme of the TENG: PET, PVC, transparent conductive electrode, and rice paper. Reprinted with permission from Ref. [90]. Copyright (2019) Published by Elsevier Ltd. **c** Electrical output of BD-TENG encapsulated in PVA. (**c1, c2**) Photograph of the implant site of PVA-coated BD-TENG. (**c1**) Right after implantation. (**c2**) 72 h after implantation. Reprinted with permission from Ref. [88]. Copyright (2016) AAAS. **d** *In vivo* exhibition of T^2^ENG’s multiple functions, such as total biodegradation, energy harvesting, biomechanical sensing, and medical therapy. Reprinted with permission from Ref. [96]. Copyright (2018) Wiley-VCH Verlag GmbH & Co. KGaA, Weinheim

As shown in Fig. 4a, all polymeric components of BN-TENGs were manufactured from natural products, such as cellulose, chitin, silk fibroin (SF), rice paper (RP), and egg white (EW). Cellulose can be prepared from wood and cotton; chitin can be extracted from shells of crabs and shrimps; RP is usually obtained from wheat, corn, and rice; and EW and SF can be collected from eggs and cocoon, respectively. The as-prepared BN-TENG consisted of natural bioresorbable polymers (NBPs) and magnesium (Mg) electrodes with a vertical contact-separation mode (Fig. 4a). Any two different NBPs (NBP1 and NBP2) from chitin, cellulose, SF, RP, and EW could act as friction layers, and the ultrathin Mg films served as counter electrodes. The obtained nanostructure arrays were dense and homogeneous, with an average roughness of about 100 nm (Fig. 4a). Two NBP spacers were incorporated between the friction layers to effectively separate them. SF films were designed as encapsulation layers to protect the BN-TENG from the external environment. The maximum electric voltage, current, and power density reached was 55 V, 0.6 µA, and 21.6 mW m^−2^, respectively (Fig. 4a). The operation time of BN-TENGs in vitro and in vivo was controlled by the encapsulation of the silk fibroin film and could last from days to weeks. After its performance in Sprague–Dawley rats, this BN-TENG was fully degraded and resorbed, avoiding a second surgical procedure and other potential side effects. Using the proposed BN-TENGs as an electric voltage source to power an electric stimulation system in vitro, the function of isolated dysfunctional cardiomyocyte clusters was successfully controlled. The beating rates of cardiomyocyte clusters were accelerated, and the consistency of the cell contraction was improved. This solution provides a new approach to treat some heart diseases, such as bradycardia and arrhythmia.

Recently, paper-based electronics has been introduced [90, 91] and since then, a significant importance was given to this topic. A novel rice paper-based biodegradable triboelectric nanogenerator (RP-TENG) was proposed [90], which is based on a single-electrode working mode (Fig. 4b). The biodegradable materials, such as rice paper and transparent conductive ink, were used as the triboelectric pairs and conductive electrode, respectively. Experimental tests yielded the values of open-circuit voltage (*V*_oc_), short-circuit current (*I*_sc_), and output power density of 244 V, 6 μA, and 37.64 μW cm^−2^, respectively. In addition, this fabricated RP-TENG was highly stable and recyclable. Finally, the electric energy generated by the RP-TENG could supply up to 45 commercial LEDs simultaneously. Further, the simple printer-paper TENG was fabricated by interlocking Kirigami patterns with maximum *V*_oc_ of 115.49 V and Q_tr_ of 39.87 nC [92].

A single-electrode lightweight TENG (E-TENG) using only edible materials was reported by Khandelwal et al. [93]. Laver coated with an edible silver leaf was used as the active film, and a rice sheet was selected as for the substrate. The surface potential, morphology, and roughness were analyzed. The laver was found triboelectrically active. The device was reabsorbed in phosphate-buffered saline (PBS) and gastric acid. The output performance was tested using paper, tissue paper, polyvinyl chloride (PVC), and fluorinated ethylene propylene (FEP). A FEP-laver E-TENG performed the best performance: electric voltage of 23 V and current of 315 nA. These electric signals were used to power several devices, such as hygrometers, wristwatches, green light-emitting diodes (LEDs), and ultraviolet (UV) LEDs.

Another highly promising material which is biocompatible, provides high mechanical strength and has a unique structure is spider silk [94–96]. Zhang et al. [97] have demonstrated the potential of a TENG based on recombinant spider silk. The authors genetically engineered the triboelectric properties of spider silk and used water lithography to further modify the output performance. The surface modification by inkjet printing allowed both the fabrication of the rough surfaces and the transport of the doping agent (molecules, nanoparticles, and drugs). As a result, this TENG was able to provide a high power density of 5290 mW m^−2^ with 47.3% efficiency.

## Application of Eco-Friendly and Biocompatible TENGs

Eco-friendly and biocompatible TENGs were mainly used to harvest electrical energy from the ambient. Various devices have been created and tested in a real environment. For example, the combination of fresh Hosta leaf with PMMA film demonstrated the possibility of designing TENGs based on structures that include leaves for both temperature sensing and powering of LEDs [62]. This kind of application shows that these TENGs hold potential for energy harvesting and to reuse polymers, which is an important advantage as polymers cannot be completely recyclable. Compatibility of TENGs with human skin allows their application as flexible devices working in a direct contact with the human body. Yang et al. [91] have also shown this ability for such kind of harvesters. Their paper-based TENG is air-permeable and washable, which is required for the contact with human skin.

Since most of natural materials are biocompatible, it is possible to use them for biosensors and monitoring of human biosignals. The function of isolated dysfunctional cardiomyocyte clusters was successfully regulated using silk fiber and magnesium films [89]. The in vivo and in vitro tests have shown a significant performance decrease from this implantable TENG during 24-h operation. Similar experiments have shown that poly(L-lactide-co-glycolide) (PLGA) and poly(caprolactone) (PCL)-based TENG are not functional after 72 h upon implantation in rats (Fig. 4c) [88]. Such kind of implantable devices with customized service time provides broad opportunities for therapy and disease diagnostics. Moreover, implantable TENG based on silk fibers can be used for drug delivery and epilepsy attack monitoring (Fig. 4d) [96]. Biocompatibility of silk fibers suggests their use as antibacterial patch [97]. The in vivo experiments have already demonstrated the antibacterial rate of 67.4% for bacteria *Staphylococcus aureus* by analyzing the infected wound throughout 7 days.

## Overall Performance of TENGs Based on Natural Materials

**Fig. 5:**
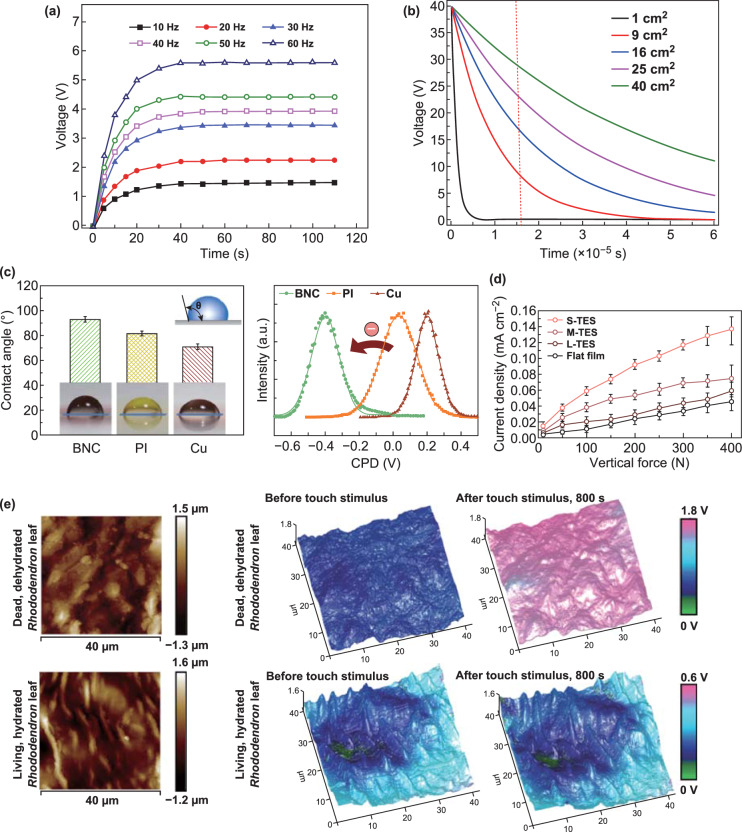
Factors influencing the output performance of TENGs: **a** influence of the excitation frequency. **b** The output voltage as a function of the size contact area of TENGs. Reprinted with permission from Ref. [82]. Copyright (2016) Published by Elsevier Ltd. **c** Static water contact angle measurement and contact potential difference comparison of materials under friction. Reprinted with permission from Ref. [78] Copyright (2017) Published by Elsevier Ltd. **d** Influence of the vertical force on output voltage. Reprinted with permission from Ref. [86]. Copyright (2016) Wiley-VCH Verlag GmbH & Co. KGaA, Weinheim; **e** Kelvin probe force microscopy of the leaf before and after friction. Reprinted with permission from Ref. [61] Copyright (2018) Wiley-VCH Verlag GmbH & Co. KGaA, Weinheim

Table 1 presents a broad range of studied triboelectric pairs based on natural leaves, plant materials, cellulose, and polymers and their performance. Analyzing the electric characteristics, one can conclude that the output voltage and current are influenced by material’s architecture and are strongly dependent on the measurement conditions. This fact suggests that the triboelectric effect is an intrinsic property of plants (from leaf to cellulose) and rigorous studies are needed to understand this phenomenon. Moreover, a unification of the test standards is required, so that the operation mode (contact-separation, single electrode, etc.) should follow similar methods to analyze different materials. Several studies demonstrated the importance of the measurement unification to identify TENGs mechanisms [29, 98, 99]. Unfortunately, many studies reported experimental tests in which TENGs were analyzed under different mechanical excitations (applied force and frequency) and size of samples (contact area), therefore not allowing an easy comparison. However, the energy characteristics are strongly dependent on the size and mechanical excitation (Fig. 5a, b, d). Different mechanical excitations used to study different TENGs perform more complex analyses. For example, the petal rose [60] and *Rhododendron* leaf [61] are able to provide 24 µW and 15 µW cm^−2^ for mechanical excitations of 2 Hz, 100 N, and 10 Hz, 0.9 N, respectively (Table 1). These observations emphasize highly nonlinear behavior of TENGs and demand establishing a standard for their testing. Following this approach, Jie et al. [62] investigated different leaves excited under the same conditions. By designing TENGs with the same area (8 × 8 cm^2^), they obtained the output electric power ranging from 180 to 2185 µW (Table 1) in different samples. Such experimental tests allow to understand the variations on the triboelectric behavior for different systems but demand deeper analyses of the mechanisms behind their excellent electric performances. It was shown that the performance of TENGs is not directly proportional to the device surface area. For example, increasing the device surfaces from 1 to 40 cm^2^ using cellulose nanofibrils leads to the increase in electric power from 35 to 1148 µW [82]. It is well known that the nonlinear dependence of power density and *I*_sc_ in relation to the surface area can be explained by the surface inhomogeneity and edge effects. All these results demonstrated the ability of various natural materials to be used as elements in the TENGs architecture. It is worth noting that the polymers were always used as a supporting material (triboelectric pair or substrate). As such, polymers are nowadays required for advanced energy harvesting technologies: Their alloys are able to design customized forms of friction bodies [91, 100–102] and to fabricate multilayer devices by different technologies providing uniform samples [74, 103–105]. Limitations related to the ability of TENGs for eco-friendly harvesting and to be fully recycled are still unsolved and require more investigations and ecological tests. Indeed, the existing polymer materials can provide high performance and be reused, even if they are not fully recyclable. As biocompatible polymers allow to fabricate flexible devices, they can be applied as intracorporeal harvesters as well [88, 106, 107]. Importantly, for many of these applications, storage supercapacitors and conditioning circuits should be used together with implantable harvesters [108] considering the architecture of the biomedical systems [109]. Moreover, the investigation of different materials is demanded for detailed studies focused on the mechanism of triboelectric phenomenon, so that a general theory can be developed to explain and to predict properties, efficient combination of materials and performance. Besides, research efforts also demonstrate the possibilities of natural materials, which can be compared with inorganic materials in terms of electric performance [110, 111]. Additionally, natural materials can be used to mimic the design of architectures based on surface modification to enhance the triboelectric effect. Despite there are many reports focused on the design of TENGs, the rational selection of pair materials has not yet been achieved, highlighting the exhaustive comparison of harvester performances. Furthermore, the mechanism of charge formation is not well understood. For this reason, some studies provided additional characterization of materials [61, 78, 82]. Figure 5c demonstrates additional measurements of the contact angle. These studies allow to identify both wetting properties of the materials and their polarity: By increasing the contact angle, the polarity is shifted to a negative charge. The contact angle is a macroscopic measure of wettability, which additionally characterizes the triboelectric material before analyzing the performance of TENGs. In this context, it is worth noting that Kelvin probe force microscopy can be also used to characterize polar properties. Figure 5e shows an example of the charge distribution on the TENG surface before and after the touch stimulus. This is a relevant experiment as it allows to investigate the mechanism of charge transfer in triboelectric effect and the influence of dehydration in a leaf during friction [61]. These measurements demonstrate that leaf drying decouples the actual energy conversion site, the cuticle, from the charge transport circuit that exists in the hydrated living tissue.

## Conclusions

This review demonstrates the potential of the natural materials in energy harvesting systems. Investigation of the TENGs and combination of different kinds of materials allow to foster the fundamental knowledge, materials application, and their characterization methods. By this way, new energy harvesting systems can be applied in a safe manner for the environment. Using various materials around us, we can create eco-friendly, biocompatible, and highly efficient devices. The overviewed literature highlights that a wide range of materials, harvester architectures, and investigation methods were already proposed. These methods show how the harvesting efficiency is influenced by the pair material, contact area, thickness, and mechanical excitation (force and frequency), etc. The novel nanotechnological methods allow a deeper understanding of the charge formation, friction materials dependence, and triboelectricity at the nanoscale. New testing methods for TENG characterization are being implemented to achieve these goals. Various methods under investigation will most likely allow to understand the charge formation and the mechanism of the triboelectric phenomenon in the forthcoming years. Using natural materials incorporated within TENGs will propel the development of innovative energy harvesting systems fully recyclable, eco-friendly, and biocompatible.
